# *Candida albicans* Possesses Sap7 as a Pepstatin A-Insensitive Secreted Aspartic Protease

**DOI:** 10.1371/journal.pone.0032513

**Published:** 2012-02-27

**Authors:** Wataru Aoki, Nao Kitahara, Natsuko Miura, Hironobu Morisaka, Yoshihiro Yamamoto, Kouichi Kuroda, Mitsuyoshi Ueda

**Affiliations:** 1 Japan Society for the Promotion of Science, Sakyo-ku, Kyoto, Japan; 2 Division of Applied Life Sciences, Graduate School of Agriculture, Kyoto University, Sakyo-ku, Kyoto, Japan; 3 Industrial Technology Center, Kyoto Municipal Institute of Industrial Technology and Culture, Simogyo-ku, Kyoto, Japan; Yonsei University, Korea

## Abstract

**Background:**

*Candida albicans*, a commensal organism, is a part of the normal flora of healthy individuals. However, once the host immunity is compromised, *C. albicans* opportunistically causes recurrent superficial or fatal systemic candidiasis. Secreted aspartic proteases (Sap), encoded by 10 types of *SAP* genes, have been suggested to contribute to various virulence processes. Thus, it is important to elucidate their biochemical properties for better understanding of the molecular mechanisms that how Sap isozymes damage host tissues.

**Methodology/Principal Findings:**

The *SAP7* gene was cloned from *C. albicans* SC5314 and heterogeneously produced by *Pichia pastoris*. Measurement of Sap7 proteolytic activity using the FRETS-25Ala library showed that Sap7 was a pepstatin A-insensitive protease. To understand why Sap7 was insensitive to pepstatin A, alanine substitution mutants of Sap7 were constructed. We found that M242A and T467A mutants had normal proteolytic activity and sensitivity to pepstatin A. M242 and T467 were located in close proximity to the entrance to an active site, and alanine substitution at these positions widened the entrance. Our results suggest that this alteration might allow increased accessibility of pepstatin A to the active site. This inference was supported by the observation that the T467A mutant has stronger proteolytic activity than the wild type.

**Conclusions/Significance:**

We found that Sap7 was a pepstatin A-insensitive protease, and that M242 and T467 restricted the accessibility of pepstatin A to the active site. This finding will lead to the development of a novel protease inhibitor beyond pepstatin A. Such a novel inhibitor will be an important research tool as well as pharmaceutical agent for patients suffering from candidiasis.

## Introduction

The commensal organism *Candida albicans* exists in almost all humans as a part of the natural microflora [1]. However, once the host immunity is diminished or the balance of the normal microflora is disrupted, *C. albicans* may turn opportunistic and cause recurrent superficial or fatal systemic candidiasis. Superficial candidiasis has a widespread occurrence, and three-quarters of healthy women experience vaginal candidiasis [2], [3]. In addition, the mortality rate of systemic candidiasis is notably high because of the lack of effective diagnosis and treatment. Thus, it is vital to elucidate the virulence mechanisms of *C. albicans* and develop novel pharmaceutical agents.

The secreted aspartic protease (Sap) family, encoded by 10 *SAP* genes, has been suggested as one of the major virulence factors of *C. albicans* [1], [4], [5], [6]. *SAP* genes are differentially regulated depending on the surrounding environments [7], and of the 10 *SAP* genes, the expression of *SAP1–3* has been mainly observed in the yeast forms [8]. Compared to them, *SAP4–6* are expressed in the hyphal forms, and are related to systemic infections and evasion from the host immune system [9]. *SAP7* expression has been detected in mouse models, but not in any *in vitro* conditions, and it correlates with virulence in intravenous infections [10]. *SAP8* is transiently expressed in yeast and epithelial models [11], [12]. *SAP9* and *SAP10*, which encode glycosylphosphatidylinositol (GPI)-anchoring domains, are expressed under many conditions, and are believed to maintain cell wall integrity through the post-translational processing of cell wall proteins [13], [14], [15].

All types of *SAP* gene products are suggested to contribute to various virulence processes *in vitro*, such as adhesion, invasion, and immune evasion. For example, they degrade host defense proteins such as mucin [16], IgG [17], IgA [18], and complements (C3b, C4b, and C5) [19]. Furthermore, Sap isozymes are needed to obtain nitrogen from proteins [8], escape from macrophages [9], and to adhere to and invade epithelial tissues [20], [21].

In this study, we found that pepstatin A did not have any effect on the proteolytic activity of Sap7. It was surprising because pepstatin A generally inhibits all aspartyl proteases. We analyzed the cause of Sap7 insensitivity toward pepstatin A, and found that the core structure of Sap7 prohibited pepstatin A from accessing the active site. This study paves the way for the development of a novel, potent inhibitor that is able to inhibit all types of Sap isozymes. Such an inhibitor might be a more effective pharmaceutical agent against candidiasis.

## Materials and Methods

### Strains and media

*Escherichia coli* strain DH5α [*F* ^−^, *ΔlacU169 (φ80lacZΔM15)*, *hsdR17* (*r_K_* ^−^, *m_K_* ^+^), *recA1*, *endA1*, *deoR*, *thi-1*, *supE44*, *gyrA96*, *relA1*, *λ* ^−^] (TOYOBO, Osaka, Japan) was used as a host for DNA manipulation. *C. albicans* strain SC5314 (American Type Culture Collection) was used for isolation of the *Candida* genome. *Pichia pastoris* strain GS115 [*his4*] (Invitrogen, CA, USA) was used as a host for protein production. *E. coli* transformants were grown in Luria–Bertani media [1% (*w*/*v*) tryptone, 0.5% (*w*/*v*) yeast extract, and 1% (*w*/*v*) sodium chloride] containing 50 µg/mL ampicillin. For protein production, *P. pastoris* transformants were pre-cultivated in buffered complex glycerol media (BMGY) [1% (*w*/*v*) yeast extract, 2% (*w*/*v*) peptone, 1.34% (*w*/*v*) yeast nitrogen base *w*/*o* amino acids, (4×10^−5^)% (*w*/*v*) biotin, and 1% (*v*/*v*) glycerol, in 100 mM potassium phosphate (pH 6.0)]. For transcriptional induction, pre-cultivated transformants were grown in buffered complex methanol media (BMMY) [1% (*w*/*v*) yeast extract, 2% (*w*/*v*) peptone, 1.34% (*w*/*v*) yeast nitrogen base *w*/*o* amino acids, (4×10^−5^)% (*w*/*v*) biotin, 0.5% (*v*/*v*) methanol, and 100 mM potassium phosphate (pH 6.0)].

### Construction of plasmids encoding mutant Sap7

All the primers used in this study are presented in Table S1. In the previous study, DNA fragments encoding the *SAP4* and *SAP7* genes were cloned from the genomic DNA extracted from *C. albicans* SC5314, and were inserted into the pHIL-S1 plasmid (Invitrogen) [22]. The resulting recombinant genes were composed of the *PHO1* secretion signal sequence, the *SAP* gene, and a FLAG-tag encoding sequence. Because *C. albicans* displays alternative CUG codon usage (Ser for Leu) [23], CUG codons in the *SAP* genes were replaced by UCG codons, which encode Ser in *P. pastoris*. The resulting plasmids were named pHIL-Sap4 and pHIL-Sap7. To determine which amino acid was important for insensitivity to pepstatin A, the target amino acids of Sap7 were substituted with alanine. The *SAP* gene sequence was mutated by the QuikChange site-directed mutagenesis technique by using 2 complementary primers. To construct pHIL-Sap7Δ422–451, 2 DNA fragments encoding 19–421 and 452–588 amino acid residues of Sap7 were amplified. Then, these DNA fragments were inserted into the pHIL-S1 vector by using In-Fusion HD Cloning Kit (Clontech, CA, USA). The DNA sequences were verified using BigDye Terminator v3.1 Cycle Sequencing Kit and 310 Genetic Analyzer (Applied Biosystems, CA, USA).

### Production and purification of FLAG-tagged Sap isozymes

pHIL-Sap4, pHIL-Sap7, and pHIL-S1, a control plasmid, were digested with *Sac*I. *P. pastoris* GS115 cells were transformed with the linear plasmids by using the Frozen-EZ Yeast Transformation II kit (Zymo Research, CA, USA). The *P. pastoris* transformant was grown in BMGY medium for 48 h at 30°C. The culture medium was subsequently centrifuged at 3000 *g* for 5 min. The cells were resuspended in BMMY medium for transcriptional induction, and then grown for 24 h at 30°C. The supernatant of the culture medium was concentrated using a YM-10 filter device (Millipore, MA, USA), and the concentrated supernatant was mixed with an anti-FLAG M2 affinity gel (Sigma-Aldrich, MO, USA) and rotated for 1 h at 4°C. The gel was washed with PBS (pH 7.4) to remove non-specific proteins. FLAG-tagged Sap isozymes were eluted from the gel by using a 3×FLAG peptide (Sigma-Aldrich). The protein concentration was quantified using the Protein Assay Bicinchoninate Kit (Nacalai Tesque, Kyoto, Japan).

### SDS-PAGE, CBB staining, and western blotting

The purified Sap isozymes were separated by SDS-PAGE with or without EndoH (New England Biolabs, MA, USA) treatment in a 5%–20% gradient polyacrylamide gel. The protein bands were detected with the CBB Stain One kit (Nacalai Tesque) or western blotting using the anti-FLAG M2 monoclonal antibody-peroxidase conjugate (Sigma-Aldrich). To determine whether the 2 fragments of Sap7 were bound to each other in a non-covalent manner, Sap7 was separated by SDS-PAGE without 2-mercaptoethanol treatment and stained with CBB.

### MALDI-TOF/MS analysis and N-terminal sequencing

The protein bands detected by CBB staining were identified using a Voyager RP MALDI-TOF/MS (Applied Biosystems). Amino acid sequencing of purified Sap isozymes was carried out by the Edman degradation method on the protein sequence system PPSQ-33A (Shimadzu, Kyoto, Japan), using a Hybond-P membrane (GE Healthcare, Little Chalfont, UK).

### Measurement of proteolytic activity

To determine the proteolytic activity of the Sap isozymes, the FRETS-25Ala library (Peptide Institute, Osaka, Japan) was used as a substrate as described previously [22]. In brief, the peptide library (final concentration, 10 µM) was mixed with Sap4 as a control or Sap7 (final concentration, 3 nM or 5 nM, respectively) in 200 µL of sodium citrate buffer (50 mM; pH 6.0) at 37°C. The increase in fluorescence was kinetically measured at λ*_ex_* = 355 nm and λ*_em_* = 460 nm. One unit of proteolytic activity was defined as that increasing the fluorescence by 1 min^−1^ at optimum pH. For inhibitory assays, pepstatin A (Peptide Institute), EDTA (DOJINDO, Kumamoto, Japan), leupeptin (Peptide Institute), or phenylmethylsulfonyl fluoride (PMSF) (Peptide Institute) were mixed with the reaction solution (final concentration, 1 µM, 1 mM, 4 µM, and 200 µM, respectively).

### Protein sequence analysis

Protein sequences of Sap isozymes were obtained from the *Candida* genome database [24]. Multiple-sequence alignments were performed with CLUSTAL W [25].

### Homology modeling

A structural model for Sap7 was generated using SWISS-MODEL based on the crystal structure of Sap2 (PBD ID: 1eag) [26], [27]. The calculated Sap7 structure was verified using a Ramachandran map generated by PDBSum [28], and visualized using Pymol (DeLano Scientific; http://www.pymol.org).

### Statistics

Mean values among groups were compared using one-way factorial ANOVA, with P<0.05 being considered statistically significant. If the ANOVA was significant, *post-hoc* pair wise comparisons were performed using a Tukey's test with P<0.05 also being considered statistically significant.

## Results

### Biochemical properties of Sap7

**Figure 1:**
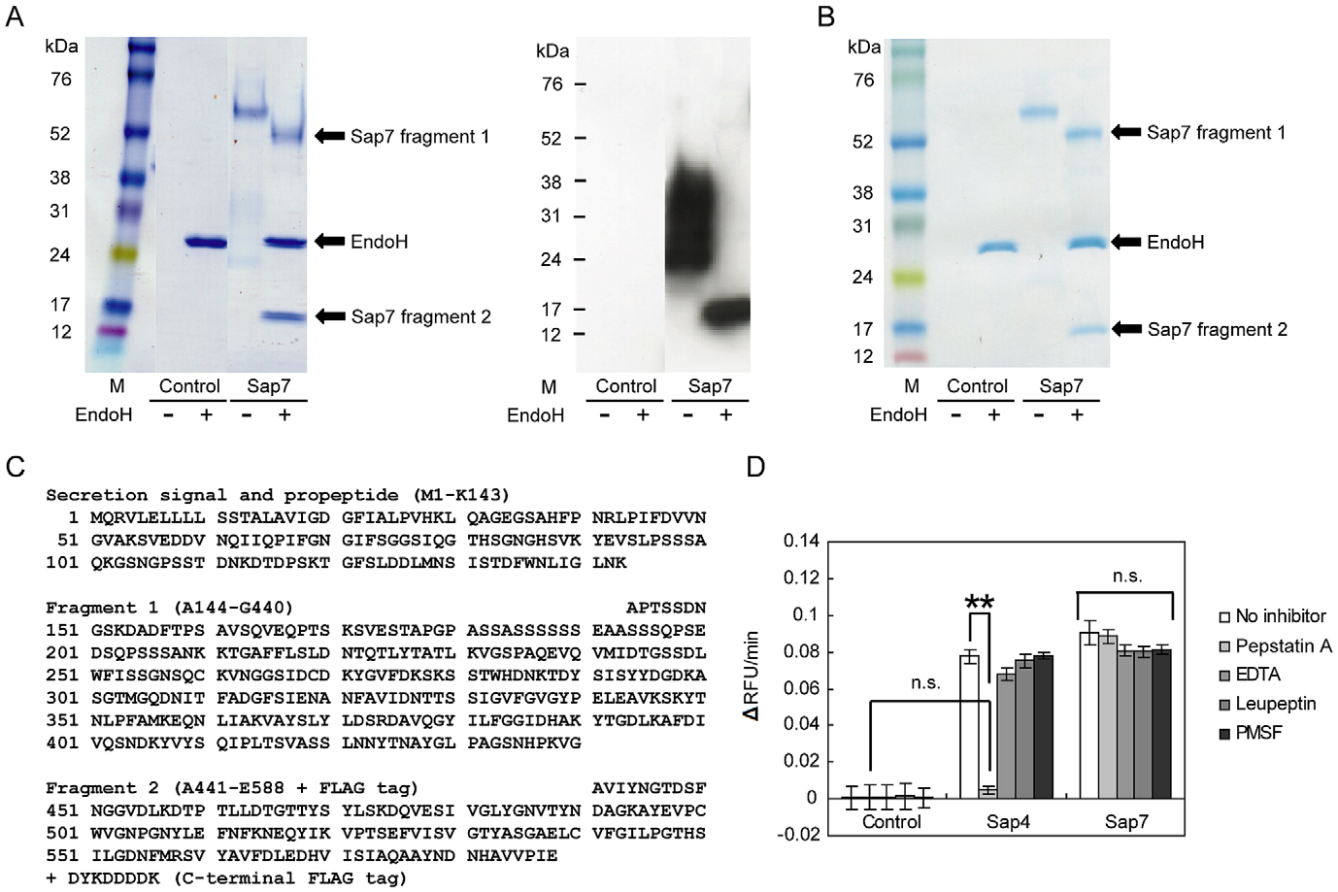
Biochemical characteristics of Sap7. (A) SDS-PAGE (left) and western blot (right) analysis of Sap7 with or without EndoH treatment. Analyses of all bands by MALDI-TOF/MS and N-terminal sequencing showed that Sap7 consisted of 2 fragments: fragment 1 (52 kDa) and fragment 2 (15 kDa). Fragment 2 was highly, heterogeneously *N*-glycosylated, as revealed by EndoH treatment and western blot analysis, which detected the FLAG-tag epitope conjugated at the C-terminal end of Sap7. M: marker, control: protein extracted from the culture supernatant of *P. pastoris* transformed with a control pHIL-S1 vector. (B) Non-reducing SDS-PAGE analysis. Electrophoretic pattern of non-reduced SDS-PAGE was the same as that of reduced, indicating that the 2 fragments interacted in a non-covalent manner. (C) Primary structure of Sap7. Sap7 was separated into 2 fragments: Fragment 1 was a 52-kDa subunit composed of A144-G440; fragment 2 was a 15-kDa subunit composed of A441-E588. (D) Sensitivity of proteolytic activity to major protease inhibitors. Proteolytic activity was measured using the FRETS-25Ala library with or without various protease inhibitors. While the activity of Sap4 was completely inhibited by pepstatin A, Sap7 did not show sensitivity to any protease inhibitors used here. Averages of at least 3 independent experiments are plotted, and the error bars show S.E.M. ** *P*<0.01 determined by the Tukey's test after a significant one-way factorial ANOVA (*P*<0.01), n.s.; not significant.

The *SAP7* gene with a C-terminal FLAG tag-encoding sequence was cloned from the *C. albicans* SC5314 genome. The DNA fragment was inserted into pHIL-S1, a vector for secretory protein production by *P. pastoris*. The transformants were cultivated in BMMY media for transcriptional induction, and the production of FLAG-tagged Sap7 was examined by SDS-PAGE and western blotting. Two distinct protein bands, a band around 60 kDa and a smear band around 24 kDa, were observed as purified protein extracts, while no band was detected in the control lane (Fig. 1A). To check whether Sap7 was glycosylated, Sap7 was mixed with EndoH that cleaves oligosaccharides from *N*-linked glycoproteins, and incubated for 1 h at 37°C. Following treatment with EndoH, these bands shifted to 52 kDa (fragment 1) and 15 kDa (fragment 2), and only the 15-kDa band was detected by western blotting using an anti-FLAG monoclonal antibody. These results indicated that Sap7 was a highly, heterogeneously *N*-glycosylated protein, and was separated into 2 distinct fragments. To determine the primary sequence of the fragments, these protein bands were excised, and analyzed by MALDI-TOF/MS and N-terminal sequencing. The analyses showed that fragment 1 was composed of amino acids from A144 to G440, and fragment 2, from A441 to E588 plus a C-terminal Flag-tag epitope (Fig. 1C). The region from M1 to K143, a secretion signal sequence and a propeptide of Sap7, was probably removed during a maturation process. In addition, non-reducing SDS-PAGE was performed to determine whether the fragments form a disulfide bond (Fig. 1B). The electrophoretic pattern of the non-reduced SDS-PAGE did not differ from that of the reduced SDS-PAGE. This result suggests that the 2 fragments of Sap7 are bound to each other in a non-covalent manner.

### Measurement of proteolytic activity of Sap7

The proteolytic activity of Sap7 was determined in 50 mM sodium citrate buffer (pH 6.0) at 37°C using the FRETS-25Ala library as a substrate. The FRETS-25Ala library contains fluorescence-quenched peptide substrates, and proteolytic activity of Sap7 was determined by measuring the fluorescence increase [29], [30]. Sap7 exhibited significant proteolytic activity, while the control showed no proteolytic activity (Fig. 1D). This result indicated that proteolytic activity was not derived from contamination by the proteases from *P. pastoris*. Surprisingly, Sap7 was not inhibited by pepstatin A and any other protease inhibitors such as EDTA, leupeptin, and PMSF. Under identical reaction conditions, the proteolytic activity of Sap4 was completely inhibited by pepstatin A (Fig. 1D).

### Identification of the active site of Sap7

**Figure 2:**
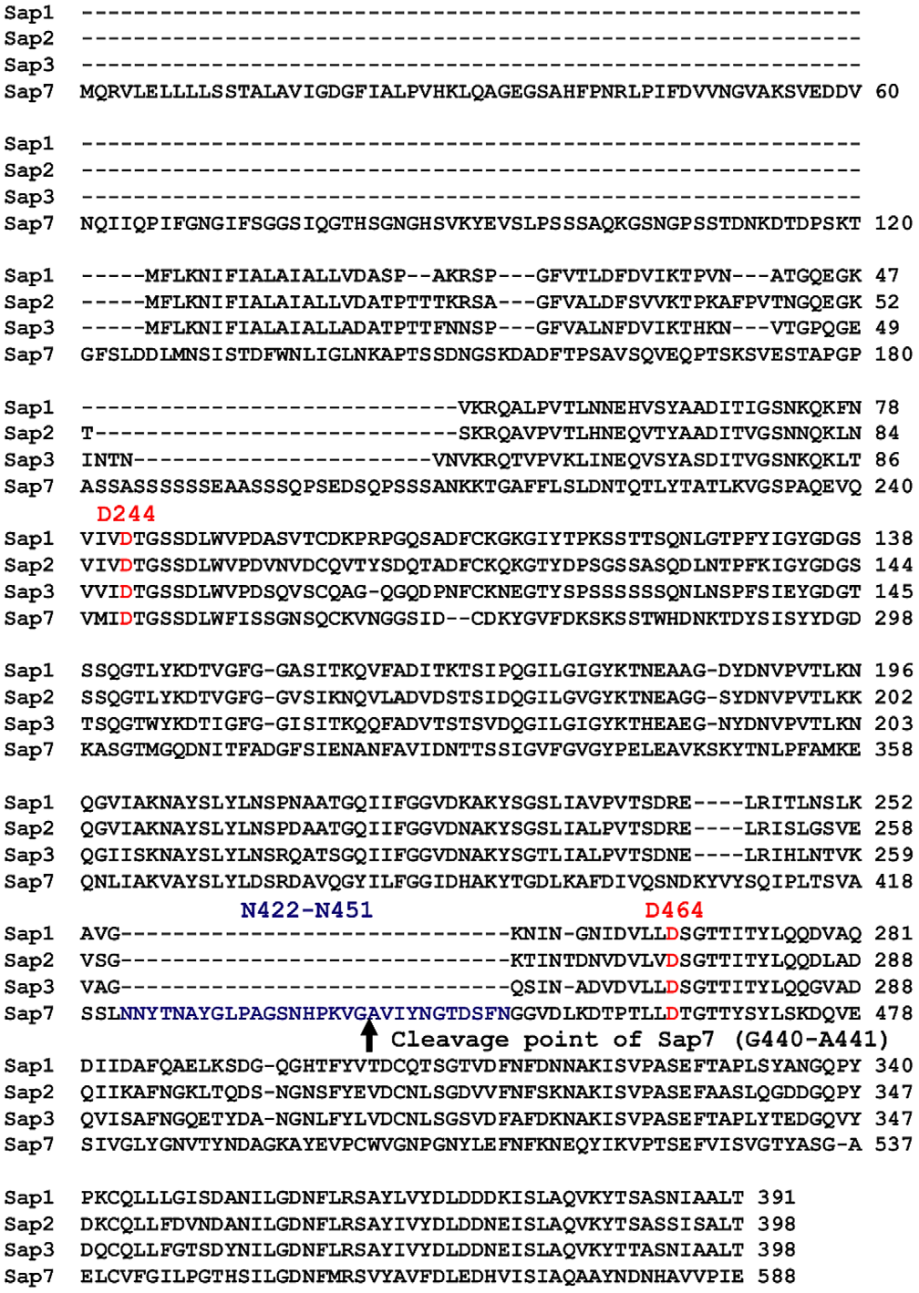
Protein sequence alignment between Sap1–3 and Sap7. Multiple-sequence alignments were performed with CLUSTAL W. The active site of Sap7 was predicted to be D244 and D464 by homology. The cleavage point of Sap7 was found in the Sap7-specific insertion sequence N422-N451, which did not exist in the sequences of Sap1–3.

**Figure 3:**
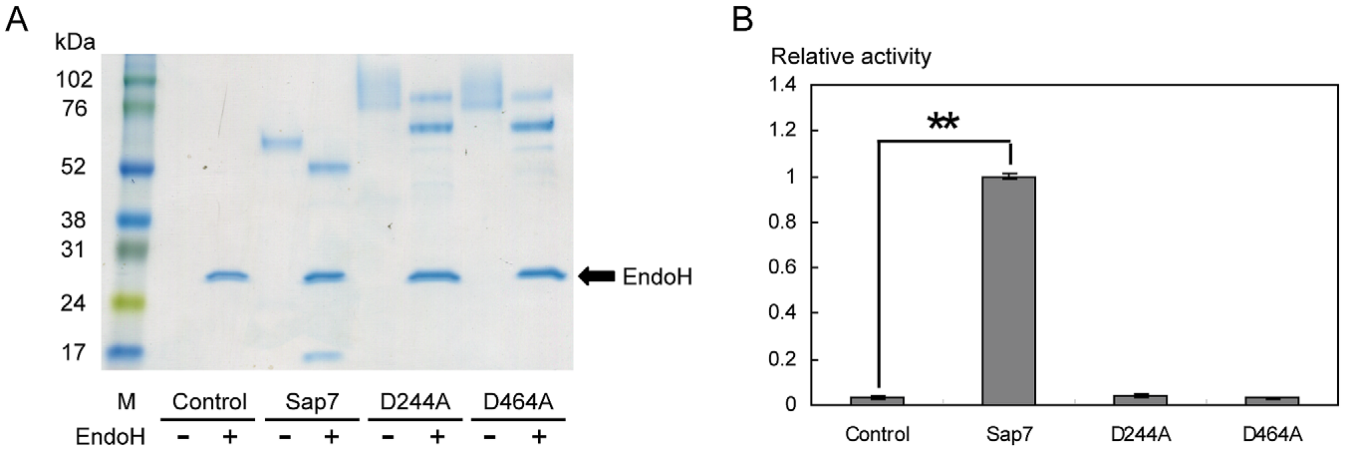
D244A and D464A mutants possess no proteolytic activity. (A) SDS-PAGE analysis with or without EndoH treatment. The D244A and D464A mutants prevented fragmentation, and lost the 15-kDa fragment found in the Sap7 wild type lane. M: marker. (B) Proteolytic activity of the mutants. The D244A and D464A mutants completely lost their proteolytic activity. This result suggests that D244 and D464 represent the active site of Sap7. Averages of at least 3 independent experiments are plotted, and the error bars are shown as ±S.E.M. ** *P*<0.01 determined by Tukey's test after a significant one-way factorial ANOVA (*P*<0.01).

To eliminate the possibility that Sap7 was not an aspartic protease, the protein sequences of Sap7 and its homologous proteases, Sap1–3, were compared using CLUSTAL W (Fig. 2). It was noted that 2 aspartic acid active site residues of Sap1–3 were also conserved in the protein sequence of Sap7. To determine whether the residues D244 and D464 formed the active site, Sap7 mutants in which these aspartic acids were substituted by alanine were constructed by the QuikChange site-directed mutagenesis technique. SDS-PAGE analysis showed that the D244A and D464A mutants lost a 15-kDa band found in the lane of wild type Sap7 (Fig. 3A). Furthermore, the mutants had no proteolytic activity (Fig. 3B). These results suggest that D244 and D464 form the active site of Sap7, and that the appearance of the 15-kDa band is caused by self-processing of active Sap7.

### Influence of *N*-glycosylation and fragmentation of Sap7 on pepstatin A-insensitivity

**Figure 4:**
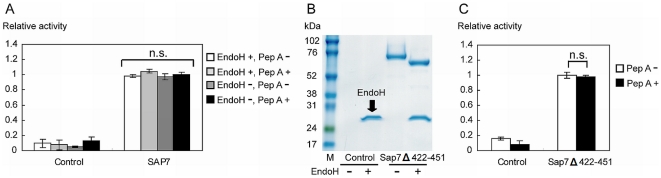
*N*-glycosylation and fragmentation of Sap7 did not affect its insensitivity to pepstatin A. (A) Proteolytic activity of deglycosylated Sap7. The influence of *N*-glycosylation on its insensitivity to pepstatin A was evaluated by EndoH treatment. Deglycosylated Sap7 did not show any significant change in proteolytic activity and pepstatin A insensitivity. (B) SDS-PAGE analysis of Sap7Δ422–451 with or without EndoH treatment. The sum of the 52-kDa and 15-kDa wild type fragments (Fig. 1A) was almost equal to the 68-kDa fragment of Sap7Δ422–451, indicating that Sap7Δ422–451 existed in a non-fragmented form. M: marker. (C) Proteolytic activity of Sap7Δ422–451. Sap7Δ422–451 was insensitive to pepstatin A. Thus, there was no relationship between the fragmentation of Sap7 and pepstatin A insensitivity. The data represent the average of at least 3 independent experiments. Error bars are shown as ±S.E.M. n.s.; not significant by Tukey's test.

We identified 2 distinctive properties of Sap7 compared to other Sap isozymes, and hypothesized that these could be responsible for pepstatin A insensitivity of Sap7. One was the highly, heterogeneously glycosylated form of Sap7. This modification of carbohydrate chains on the protein surface could act as a barrier to pepstatin A. The other was the fragmentation of Sap7. The 2 catalytic aspartic acids were separated into 2 fragments, and were found at the interface. If the interaction between these fragments was weak, pepstatin A could not form a stable bond with Sap7. To verify the first hypothesis, the protease activity of Sap7 was measured after EndoH treatment, which eliminated all *N*-glycosylation. However, we observed no significant differences in the proteolytic activity and pepstatin A-insensitivity of Sap7 with or without EndoH (Fig. 4A). This result indicated that *N*-glycosylation did not affect pepstatin A-insensitivity. For the second hypothesis, we constructed a mutant, which prevented the intramolecular fragmentation. We found that the fragmentation point of Sap7 was located at the center of a Sap7-specific sequence, N422–N451 (Fig. 1C and Fig. 2). To determine whether this sequence was required for fragmentation, a Sap7Δ422–451 mutant was constructed. SDS-PAGE analysis showed that the mutant had a single 68-kDa band after EndoH treatment (Fig. 4B); the approximate sum of the 52-kDa and 15-kDa wild type fragments (Fig. 1A). Thus, a Sap7 mutant without fragmentation was successfully constructed. On measurement, the proteolytic activity of the mutant was found to be insensitive to pepstatin A (Fig. 4C). As a result, the fragmentation of Sap7 was determined to be unrelated to pepstatin A insensitivity.

### Determination of important amino acids for pepstatin A insensitivity

**Figure 5:**
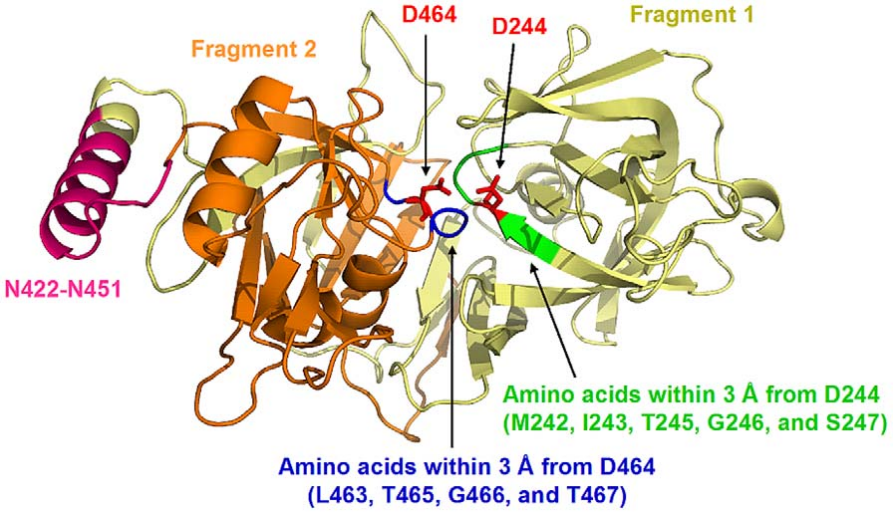
Structure of Sap7 calculated by SWISS-MODEL. The structure of Sap7 was calculated using SWISS-MODEL based on the crystal structure of Sap2 (1eag), and visualized using Pymol. Red: D244 and D464, green: amino acids within 3 Å of D244, blue: amino acids within 3 Å of D464, pale yellow: fragment 1, orange: fragment 2, and pink: N422–N451.

**Figure 6:**
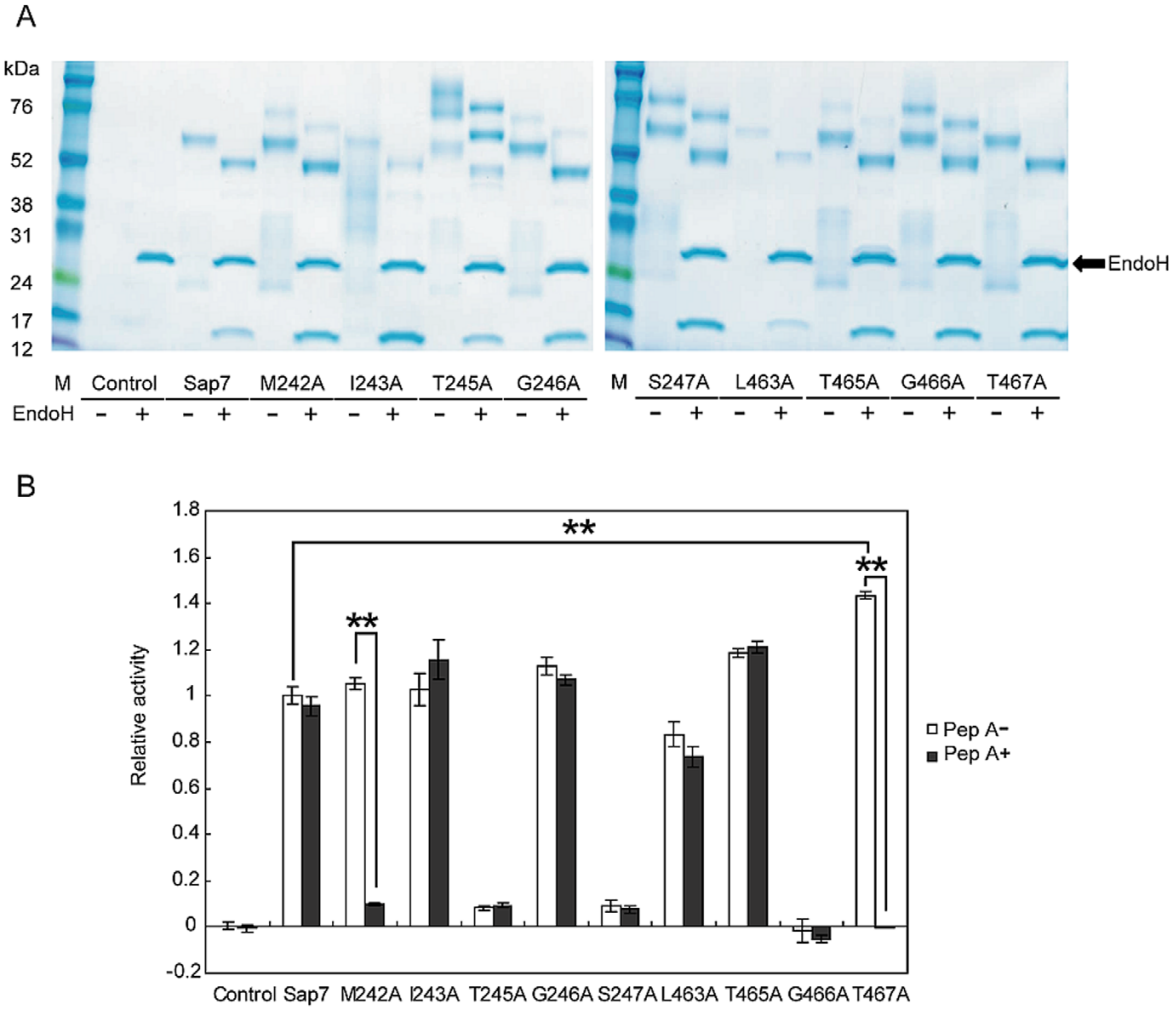
M242 and T467 are important amino acids in restricting the accessibility of pepstatin A to the active site. (A) SDS-PAGE analysis of alanine substitution mutants with or without EndoH treatment. All mutants were successfully produced by *P. pastoris*, and a 15-kDa band was confirmed in all samples. M: marker. (B) Proteolytic activity with or without pepstatin A. After pepstatin A treatment, M242A showed some proteolytic activity, while T467A showed none. Relative activity of the T467A mutant was significantly stronger than that of wild type. Average of at least 3 independent experiments are plotted, and the error bars are shown as ±S.E.M. ** *P*<0.01 determined by Tukey's test after a significant one-way factorial ANOVA (*P*<0.01).

**Figure 7:**
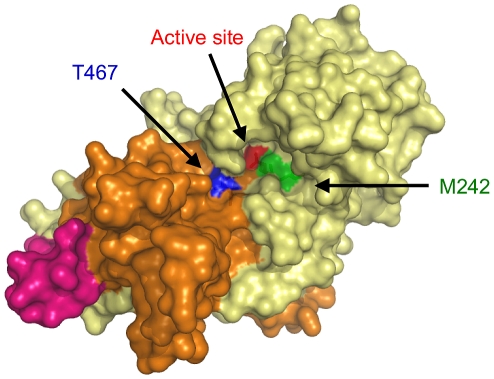
Surface modeling of Sap7. The surface structure of WT Sap7 was calculated using SWISS-MODEL based on the crystal structure of Sap2 (1eag), and visualized using Pymol. M242 and T467 were located near the entrance to the active site. Red: D244 and D464, green: M242, blue: T467, pale yellow: fragment 1, orange: fragment 2, and pink: N422–N451.

Next, we tested the hypothesis that amino acid residues in close proximity to the active site inhibited pepstatin A access. To verify this hypothesis, the tertiary structure of Sap7 was calculated using SWISS-MODEL based on the crystal structure of Sap2, which has a homologous protein sequence with Sap7 [26] (Fig. 5). In the previous section, we demonstrated that fragmentation had no effect on Sap7 activity (Fig. 4), and therefore, in the model, we calculated the tertiary structure of Sap7 as a non-fragmented protein. The calculated structure is expected to reflect the native folding of fragmented Sap7. Amino acids within 3 Å of D244 and D464 (shown in blue and green, respectively) were calculated using the Pymol program, and identified to be M242, I243, T245, G246, S247, L463, T465, G466, and T467. Each amino acid within 3 Å of the catalytic residues was substituted by alanine. All the mutants were successfully produced by *P. pastoris* (Fig. 6A), and we found that the proteolytic activities of the M242A and T467A mutants were inhibited by pepstatin A (Fig. 6B). Interestingly, T467A showed higher proteolytic activity than the wild type. M242 and T467 are located at the entrance to the active site (Fig. 7), and their side chains partially cover the entrance. As compared to the wild type, the M242A and T467A mutants had relatively wider entrances, because alanine has a side chain smaller than that of methionine and threonine. This alteration might allow pepstatin A to enter the active site.

## Discussion

In this study, we used the *P. pastoris* expression system and FLAG-tag affinity purification to purify Sap7, because *C. albicans* has never produced Sap7 *in vitro* [31]. *SAP7* is found to be expressed by *C. albicans in vivo* [32]; however, it is difficult to purify sufficient amounts of Sap7 under such conditions. The *P. pastoris* expression system enabled us to obtain large quantities of Sap isozymes for determining various enzymatic parameters. In addition, protein folding by the *P. pastoris* expression system reflects that of *C. albicans* [9], [33]. Indeed, other Sap isozymes produced by *P. pastoris* have been shown to have very similar properties to those produced by *C. albicans*.

Interestingly, in our study, Sap7 was not inhibited by pepstatin A (Fig. 1D). Pepstatin A is a potent inhibitor of almost all types of aspartic proteases, and has been used to evaluate the virulence of the Sap family [26], [34], [35]. Previously, a family of pepstatin A-insensitive acid proteases was identified, which had originally been characterized as aspartic proteases [36], [37]. These proteases are now known as glutamic proteases that use glutamic acid as the catalytic residue instead of aspartic acid. To determine whether Sap7 is actually an aspartic protease, we carried out a sequence alignment between Sap7 and Sap1–3, and found that 2 catalytic aspartic acids of Sap1–3 were also conserved in Sap7 (Fig. 2). Alanine substitution in these amino acids led to the loss of the proteolytic activity of Sap7 (Fig. 3B), indicating that Sap7 is not a glutamic protease, but an aspartic protease. Thus, we propose that the pepstatin A insensitivity of Sap7 is because of a novel mechanism.

Our results showed that Sap7 has 2 important distinctive characteristics: high *N*-glycosylation and intramolecular fragmentation. These properties are thought not to be artifacts, but rather inherent in Sap7 itself. The mechanism of *N*-glycosylation of *P. pastoris* somewhat differs from that of *C. albicans* [38]; however, the *N*-glycosylation pattern of the proteins heterogeneously produced by *P. pastoris* is consistent with that observed in the proteins from *C. albicans* [22], [33], [39]. The intramolecular fragmentation of Sap7 was dependent on the Sap7-specific N422–N451 sequence (Fig. 2), and did not occur in the D244A and D464A mutants, which possessed no proteolytic activity (Fig. 3A). These observations indicate that fragmentation is an intrinsic property of Sap7, and not due to *P. pastoris* proteases. We assessed whether these distinct properties affected pepstatin A insensitivity, and found no correlation (Fig. 4).

Next, we proposed the tertiary structure of Sap7 using SWISS-MODEL to test the hypothesis that amino acids in close proximity to the active site inhibited pepstatin A access. We identified 9 amino acids, namely, M242, I243, T245, G246, S247, L463, T465, G466, and T467, within 3 Å from the active site (Fig. 5), and found that the M242A mutant was strongly inhibited and the T467 mutant was completely inhibited by pepstatin A (Fig. 6B). Some of the other alanine substitution mutants showed little proteolytic activity, probably because these residues were essential to form the proper active site structure required to catalyze the hydrolysis of the FRETS-25Ala library.

To understand why the M242A and T467A mutants were sensitive to pepstatin A, we calculated the surface structure of Sap7 (Fig. 7). M242 and T467 were located on the surface of the entrance to the active site, and the alanine substitution widened the entrance. This alteration might increase the accessibility of pepstatin A to the active site. Proteolytic activity of the T467A mutant was completely inhibited by pepstatin A; thus, the entrance was thought to be wider than that in wild type Sap7 and the M242A mutant. This hypothesis was supported by the observation that the T467A mutant had higher proteolytic activity than wild type Sap7, indicating easy access of the substrate to the center (Fig. 6B). T467 is a conserved amino acid between Sap1–3 and Sap7, and therefore, it was assumed that Sap7 controlled the accessibility of pepstatin A by a coupling effect between T467 and other amino acids.

Similar to Sap7, Sap9 and Sap10 are suggested to be less sensitive to pepstatin A [40]. The reason why *C. albicans* evolved Sap7, Sap9, and Sap10 as pepstatin A-insensitive proteases remains to be clarified. It is possible that they are countermeasures against natural aspartic protease inhibitors.

Many reports showed that Sap-deficient mutants attenuate virulence and administration of pepstatin A inhibits various forms of candidiasis [9], [21], [41], [42], [43], [44]. Thus, it is evident that Sap isozymes have important roles in the infectious process. However, some reports suggested that Sap isozymes had a little effect on some infection models because pepstatin A showed a little protective effect [1], [45], [46], [47], [48]. Pepstatin A-insensitive Sap isozymes including Sap7, Sap9, and Sap10 may have a partial role to compensate for the reduced activities of other Sap isozymes by pepstatin A.

In conclusion, we found that Sap7 was a pepstatin A-insensitive protease, and that M242 and T467 restricted the accessibility of pepstatin A to the active site. Based on the calculated structure of pepstatin A-insensitive Saps, a novel protease inhibitor capable of inhibiting the whole Sap family could be designed.

## Supporting Information

Table S1 **Primers used in this study.** The desired mutation is shown in red characters in the mutagenic oligonucleotide primers.
(XLS)
